# Primo Vascular System Accompanying a Blood Vessel from Tumor Tissue and a Method to Distinguish It from the Blood or the Lymph System

**DOI:** 10.1155/2013/949245

**Published:** 2013-05-12

**Authors:** Jaekwan Lim, Sungwoo Lee, Zhendong Su, Hong Bae Kim, Jung Sun Yoo, Kwang-Sup Soh, Sungchul Kim, Yeon Hee Ryu

**Affiliations:** ^1^Nano-Primo Research Center, Advanced Institutes of Convergence Technology, Seoul National University, Suwon 443-270, Republic of Korea; ^2^Chinese Traditional Veterinary Laboratory, Department of Animal Medicine, Northeast Agriculture University, Harbin 150-030, China; ^3^Center of Amyotrophic Lateral Sclerosis, Wonkwang University Hospital, Gwangju 503-310, Republic of Korea; ^4^Department of Acupuncture, Korea Institute of Oriental Medicine, Daejeon 305-811, Republic of Korea

## Abstract

A primo vessel was observed in the abdominal cavity in the lung cancer mouse model, and its function as an extra metastatic path was observed. In this work, we found a primo vessel accompanying a blood vessel emanating from a tumor in the skin. We also presented simple and efficient criteria to distinguish a primo vessel from a blood or a lymph vessel and from a nerve. The criteria for using DAPI and Phalloidin will be useful in clinical situations to find and identify the primo vessels among the blood vessels, lymph vessels, or nerves in the tissue surrounding a tumor such as a melanoma or breast cancer.

## 1. Introduction

Primo vessels were recently reported as additional paths of cancer metastasis besides blood or lymph vessels [1–3]. They were originally found as a novel circulatory system corresponding to acupuncture meridians [4]. Even though primo vessels accompanying blood vessels were expected from the general theory of primo vessels [5], none had previously been observed in the area of a tumor.

In this paper, we present a simple method to observe primo vessels accompanying blood vessels emerging out of tumor tissue by using a staining technique with Phalloidin and 4′, 6-diamidino-2-phenylindole (DAPI). This method was effectively used to distinguish primo vessels from blood vessels or lymph vessels in the mesentery of mice [6] and was partially used in earlier work [3]. Phalloidin shows the F-actin distribution of cells, and DAPI reveals the shape of the nuclei. Blood, lymph, and primo vessels turn out to show distinctive patterns in Phalloidin and DAPI images [3, 6]. We specifically studied the primo vessels and the blood vessels in the myofascia under the hypodermis around a tumor that was xenografted into the dorsal skin of a nude mouse.

A primo vessel is transparent and too thin to be detected with a stereomicroscope. Ordinary histological examination with hematoxylin and eosin is not effective in revealing this novel structure, because the structure is filled with collagenous fibers and is indistinguishable from the surrounding connective tissue. Therefore, our technique should be very useful for detecting this elusive novel conduit of cancer metastasis. It is especially convenient to distinguish a primo vessel from lymph vessels without having to use the time-consuming immunohistochemical method of LYVE-1 to make the distinction, which was essential in previous work [3].

## 2. Materials and Methods

### 2.1. Cell Culture

NCI-H460 human lung cancer cells were purchased from the Korean Cell Line Bank (Seoul, Republic of Korea). Cancer cells were cultured in a RPMI-1640 medium (GIBCO, USA) supplemented with 1% penicillin-streptomycin and 10% fetal bovine serum (GIBCO, USA). Cancer cells were incubated in 95% air and 5% CO_2_ at 37°C.

### 2.2. Animal Cancer Model

Female athymic nude mice (BALB-c-nu/nu, 5 weeks, weight = 15–20 g; DooYeol Biotech, Seoul, Republic of Korea) were used. The mice were inoculated subcutaneously in the dorsal skin with 2 × 10^6^ NCI-H460 human lung cancer cells (in a 0.2-mL RPMI-1640 medium) to form tumors under the skin. All research involving the animals was approved by the Institute of Laboratory Animal Resources of Seoul National University.

### 2.3. Method to Find a Primo Vessel around a Tumor

After four to eight weeks of inoculation of the cancer cells, the mouse was anesthetized using a Zoletil/Rompun intraperitoneal (IP) injection. The epidermis, dermis, and hypodermis around the tumor tissue were incised carefully at 3~5 mm from the boundary of the tumor tissue under a stereomicroscope (SZX12, Olympus, Japan). We tried to find a primo vessel along the blood vessel, but it was almost impossible to find a primo vessel under the stereomicroscope without any special treatment. The distribution of primo, blood, and lymph vessels and nerves was photographed with a CCD camera (DP70, Olympus, Japan). Several parts of primo, blood, and lymph vessels and nerves in the myofascia around the tumor tissue were taken and fixed in 10% neutral buffered formalin (NBF) for 1 hour to prevent DNA from flowing out of the nuclei during long Phalloidin treatment. After fixation, samples were stained with Alexa Fluor 568 Phalloidin (Molecular Probes, USA) for more than 15 hours and were then treated with Prolong Gold Antifade Reagent with DAPI (Molecular Probes, USA) for 2 hours to counterstain for nuclei. Finally, samples were mounted with a cover slip and photographed with a phase-contrast fluorescent microscope and a CCD camera with the inscribed scale bar. The diameters of the blood vessel, lymph vessel, nerve, and the primo vessel were measured with the scale bar. 

## 3. Results

**Figure 1:**
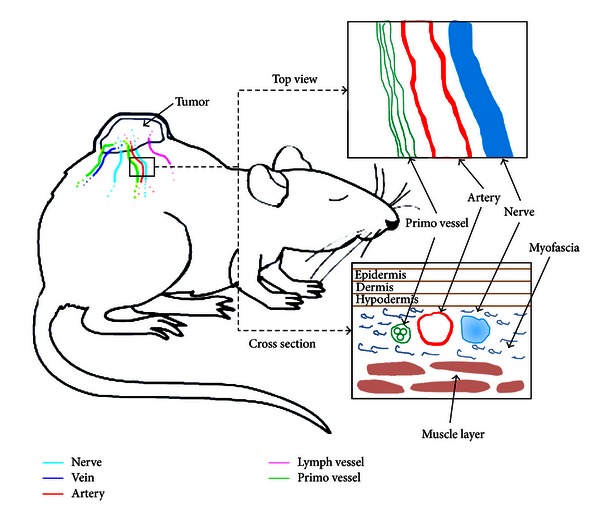
Illustration of primo vessels observed along a blood vessel. A primo vessel was found with a blood vessel or a nerve bundle inside the myofascia of the skin. The surrounding tissue around a xenografted tumor in the skin of the dorsal lumbar area (the boxed region near the tumor) was illustrated with the top view and the cross-sectional view. The nerve, artery, and primo vessel in the myofascia on the muscle layer were shown by arrows. The myofascia became thicker and squishier compared to normal skin.

As illustrated in Figure 1, well-developed blood vessels, lymph vessels, primo vessels, and nerves emanated from the tumor tissue on the dorsal skin of a nude mouse. We studied these vessels and nerves in the myofascia covering the muscle layer under the skin.

**Figure 2:**
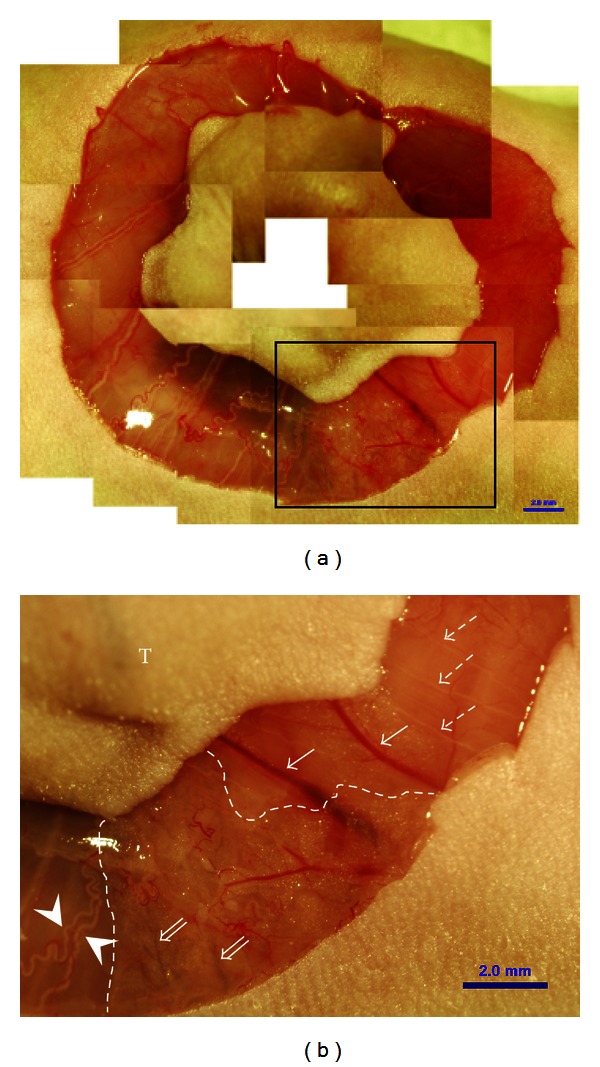
Stereoscopic image of a tumor and its surrounding area. (a) The skin was incised, and blood, lymph, and nerves in the myofascia were exposed. The rectangular area is magnified in (b). (b) Blood vessels (arrows), lymph vessels (double arrows), nerves (dotted arrows), and bundles of blood vessels and nerves (arrowheads) are seen connected to tumor tissue. They came out from the tumor (T) in radial direction. A lymphatic network that develops in adipose tissue (area between the dotted lines) can also be seen. Primo vessels accompanying blood vessels were not detectable with a stereomicroscopic image.

The skin around the tumor was incised, and the myofascia layer was exposed, as shown in the stereomicroscopic image in Figure 2(a). The rectangular area in Figure 2(a) was magnified (Figure 2(b)) to reveal the blood vessels (*arrows*), lymph vessels (*double arrows*), nerves (*dotted arrows*), and a bundle of blood vessels and nerves (*arrowheads*). Primo vessels were not detectable with a stereomicroscope.

As shown in Table 1, we studied six mice and observed the primo vessels accompanying blood vessels or bundles of blood vessels and nerves. The diameters of the primo vessels were rather uniform and were in the range of 10–20 *μ*m.

**Figure 3:**
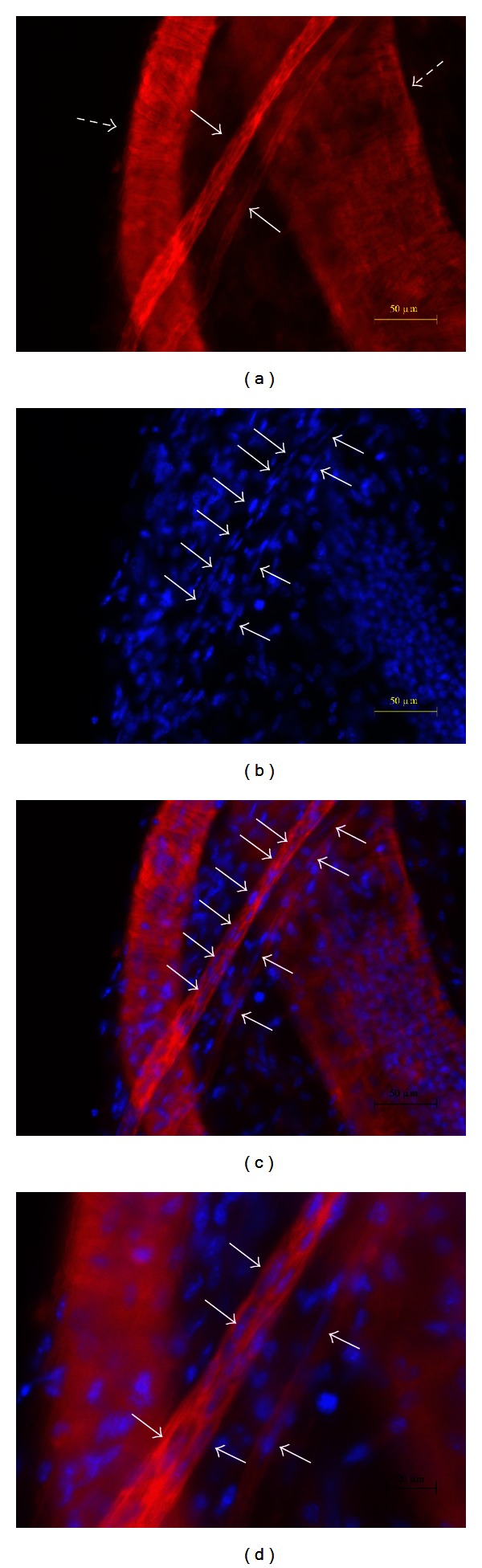
Images of blood vessels with accompanying primo vessels stained with DAPI and Phalloidin. (a) Fluorescent images of primo vessels (arrows) and blood vessels (dotted arrows) stained with Phalloidin. Blood vessels have a smooth muscle structure, whose Phalloidin signal pattern of F-actins had many transversal components to the blood vessel direction. The pattern of F-actins of the two primo vessels (arrows) was only longitudinal and therefore distinct from the patterns of the blood vessels. (b) Fluorescent image of the same sample as that in (a) with DAPI staining of nuclei. Primo vessels have rod-shaped nuclei (arrows). (c) A merged image of (a) and (b). (d) A magnified view of (c). Rod-shaped nuclei (arrows) are clearly seen in the primo vessels which are demonstrably different from the nearby blood vessels.

The DAPI and Phalloidin techniques [6], which can distinguish a primo vessel from a blood or a lymph vessel, were applied to detect the primo vessel, as shown in Figure 3. The blood vessels (*dotted arrows*) showed transverse patterns, whereas the primo vessels (*arrows*) had longitudinal patterns of Phalloidin stains (Figure 3(a)). In addition, the longitudinal alignment of the rod-shaped nuclei in the primo vessels is a clear indicator to distinguish a primo vessel from a blood vessel, as shown in Figures 3(b), 3(c), and 3(d). The rod-shaped nuclei (*arrows*) are distinct in the magnified view (Figure 3(d)) of the merged image of the Phalloidin and the DAPI signals (Figure 3(c)).

**Figure 4:**
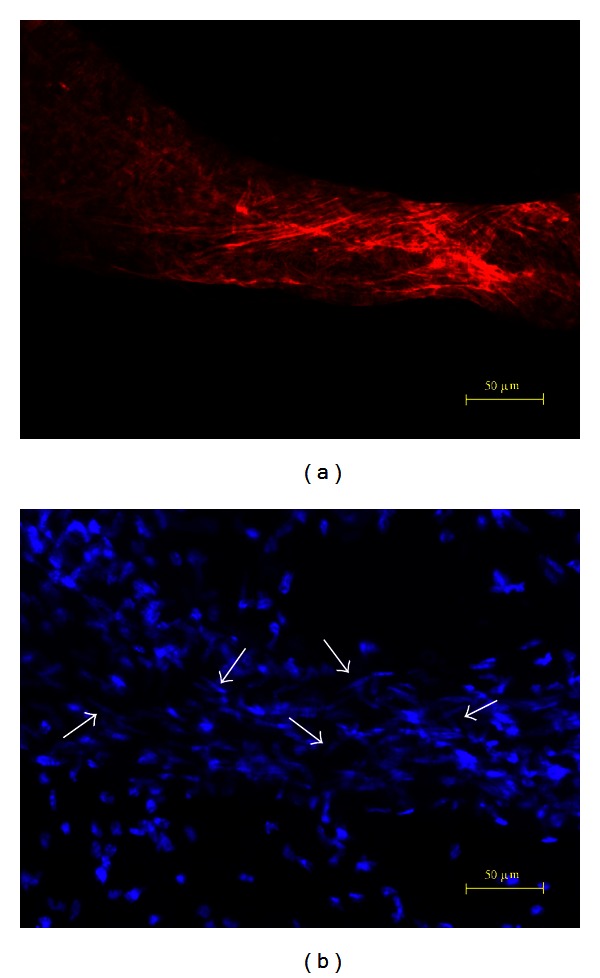
DAPI and Phalloidin images of a lymph vessel. The patterns of red-stained F-actins shown by Phalloidin (a) and blue-stained nuclei shown by DAPI (b) of a lymph vessel were irregular and readily distinguishable from blood vessels or primo vessels.

**Figure 5:**
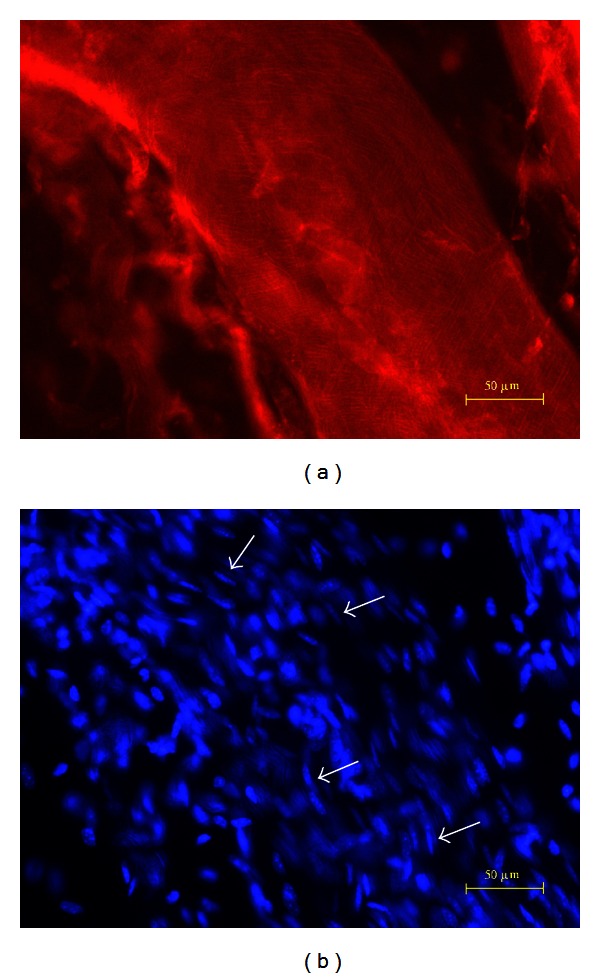
DAPI and Phalloidin images of a nerve. The F-actin distribution (a) of the nerve is much less than that of blood or primo vessels. The nuclei distribution is denser than that of primo vessels. Nuclei (blue) are of various shapes and scattered around, while the nuclei of a primo vessel is mainly rod-shaped and arranged in broken lines.

The Phalloidin and the DAPI images of a lymph vessel are presented in Figure 4. The Phalloidin pattern and irregular nuclei distribution are manifestly different from those of blood and primo vessels. Nerves showed less F-actin distribution (Figure 5(a)) and some longitudinally distributed nuclei (Figure 5(b)). Nerves can be easily distinguished from blood, lymph, or primo vessels with a stereomicroscope.

The current work did not provide immunofluorescence proof that the primo vessel was different from blood or lymph vessels, because this was already provided in previous work [2, 3]. Nevertheless, for reader convenience, we provided a CD31 and LYVE-1 test of the primo vessels, arteries, veins, and lymph nodes as in the Supplementary Information (see Figures S1 and S2 available online at http://dx.doi.org/10.1155/2013/949245).

## 4. Discussion

The purpose of this study was to present simple and efficient criteria to discern primo vessels from blood vessels, lymph vessels, and nerves in the myofascia under the skin around a tumor. In previous work [1–3], primo vessels were observed in the abdominal cavity or on the surface of the hypodermis of a xenografted tumor in a mouse or rat but not in the pathologically developed, thick, and squashy myofascia that developed around the tumor. The rationale is based upon our previous work of immunofluorescence analysis verifying the primo vessels amongst blood vessels and lymph vessels [2, 3].

We confirmed the existence of primo vessels near the adventitia of blood vessels emerging from tumor tissue. These primo vessels could be distinguished from blood or lymph vessels using simple techniques of staining with Phalloidin and DAPI and reading the resultant patterns. This new technique is different from the Trypan blue method, which until now was the only published method to find and identify the primo vessels. The Trypan blue technique is applicable only to live tissue and therefore is limited to *in vivo* situations. The current technique with Phalloidin and DAPI is applicable to tissue samples whether or not they are biologically fixed. This new technique can be useful in dealing with clinical melanoma or breast cancer situations where tissues around a tumor are available for examination of metastasis through blood, lymph, or primo vessels.

The importance of blood vessels and angiogenesis in cancer biology, especially with respect to growth [7] and metastasis [8, 9], does not need to be emphasized. However, no one has noticed the presence of another circulatory conduit near the adventitia of blood vessels. The suggested functions of the primo vessels, in general, include a path for neurotransmitter hormones [10], a circulatory path [11] for primo-fluid-containing stem-cell-like microcells [12], and proteins related to stem cell differentiation [13]. Evidence also exists for cancer metastasis through the primo vessel [2]. These functions may have some relevancy in the primo vessel accompanying a blood vessel emerging from tumor tissue. A blood vessel with accompanying primo vessels may be more active in metastasis than previously understood. 

Our research may remind readers of the discovery by Hendrix of vasculogenic mimicry [14] which is a new conduit different from blood or lymph vessels related to cancer events. This vessel was formed by a reversion of tumor cells to an undifferentiated phenotype and recapitulated embryonic vasculogenesis. Elucidating the relationship between primo vessels and vasculogenic mimicry as networks between the inside and the outside of tumor tissue remains to be done.

In conclusion criteria to find and identify by using Phalloidin and DAPI staining the primo vessels that were developed along the blood vessels emanating from a tumor tissue in the myofascia underneath mouse skin were presented. This method was based upon the different patterns of nuclei and F-actin distribution in the primo vessels, blood vessels, lymph vessels, and nerves.

## Supplementary Material

We provide the following immunofluorescence data of our unpublished work, for readers convenience, showing that the primo vessel is different from artery, vein, or lymph node. Figure S1 shows that a primo vessel is different from blood vessel with respect to the CD31 expression. A lymph node is used as a negative control. Figure S2 demonstrates that a primo vessel is different from lymph system with respect to the LYVE-1 expression. Blood vessels are used as a negative control.

## Figures and Tables

**Table 1:** Size of tumors, blood vessels, lymph vessels, primo vessels, and nerves.

Subject no.	Shape and size of tumor (cm, width × length × height)	Diameter of blood vessel (*μ*m)	Diameter of lymph vessel (*μ*m)	Diameter of primo vessel (*μ*m)	Diameter of nerve (*μ*m)	Site of primo vessel
1	Not recorded	190	60	20	120	Along BV
2	Oval shape (2.5 × 3.0 × 2.5)	40	×	20	150	Along BV
3	Irregular shape (3.5 × 2.5 × 2.0)	160	×	10	×	Along BV
4	Irregular shape (3.1 × 2.8 × 1.0)	25	100	15	110	Along BV
5	Irregular shape (2.6 × 2.4 × 1.5)	30	×	10	320	Along BBN
6	Irregular shape (not recorded)	110	×	16	390	Along BBN

×: not observed; BV: blood vessel; BBN: bundle of blood vessel-nerve.
