# Supplementary material for: Primo Vascular System Accompanying a Blood Vessel from Tumor Tissue and a Method to Distinguish It from the Blood or the Lymph System
Source: Evid Based Complement Alternat Med. 2013 May 12;2013:949245. doi: 10.1155/2013/949245 (PMC3666305; doi:10.1155/2013/949245)
Supplement: Supplementary file 1 — We provide the following immunofluorescence data of our unpublished work, for readers convenience, showing that the primo vessel is different from artery, vein, or lymph node. Figure S1 shows that a primo vessel is different from blood vessel with respect to the CD31 expression. A lymph node is used as a negative control. Figure S2 demonstrates that a primo vessel is different from lymph system with respect to the LYVE-1 expression. Blood vessels are used as a negative control. [file 949245.f1.pdf]

Supplementary Information

Immunofluorescence staining of primo vessel

Fig. 1.1 Artery: CD31

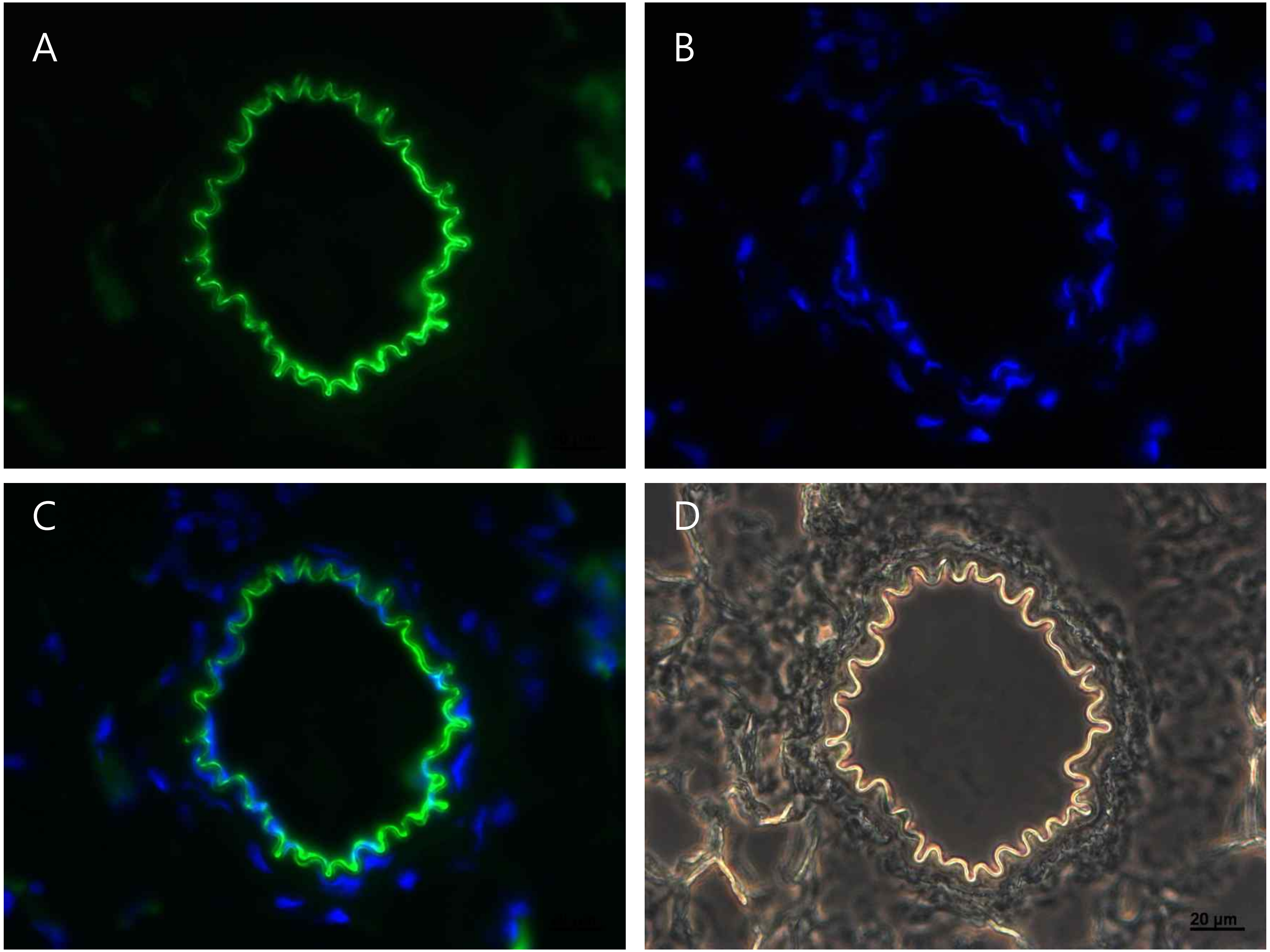

A – CD31

B – DAPI

C – CD31 +DAPI

D – Phase contrast

Fig. 1.2 Vein: CD31

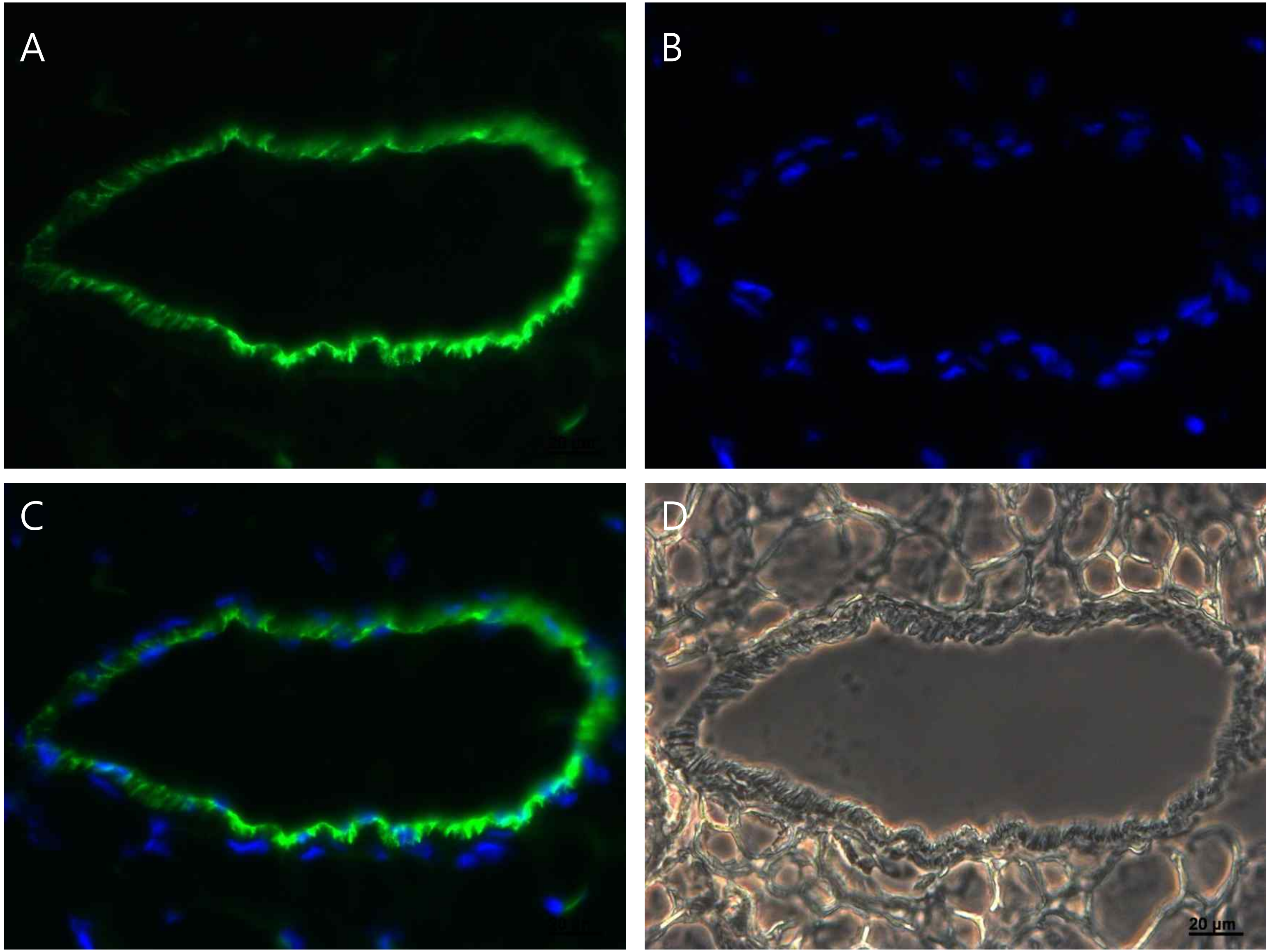

A – CD31

B – DAPI

C – CD31 +DAPI

D – Phase contrast

Fig.1.3 Lymph node: CD31

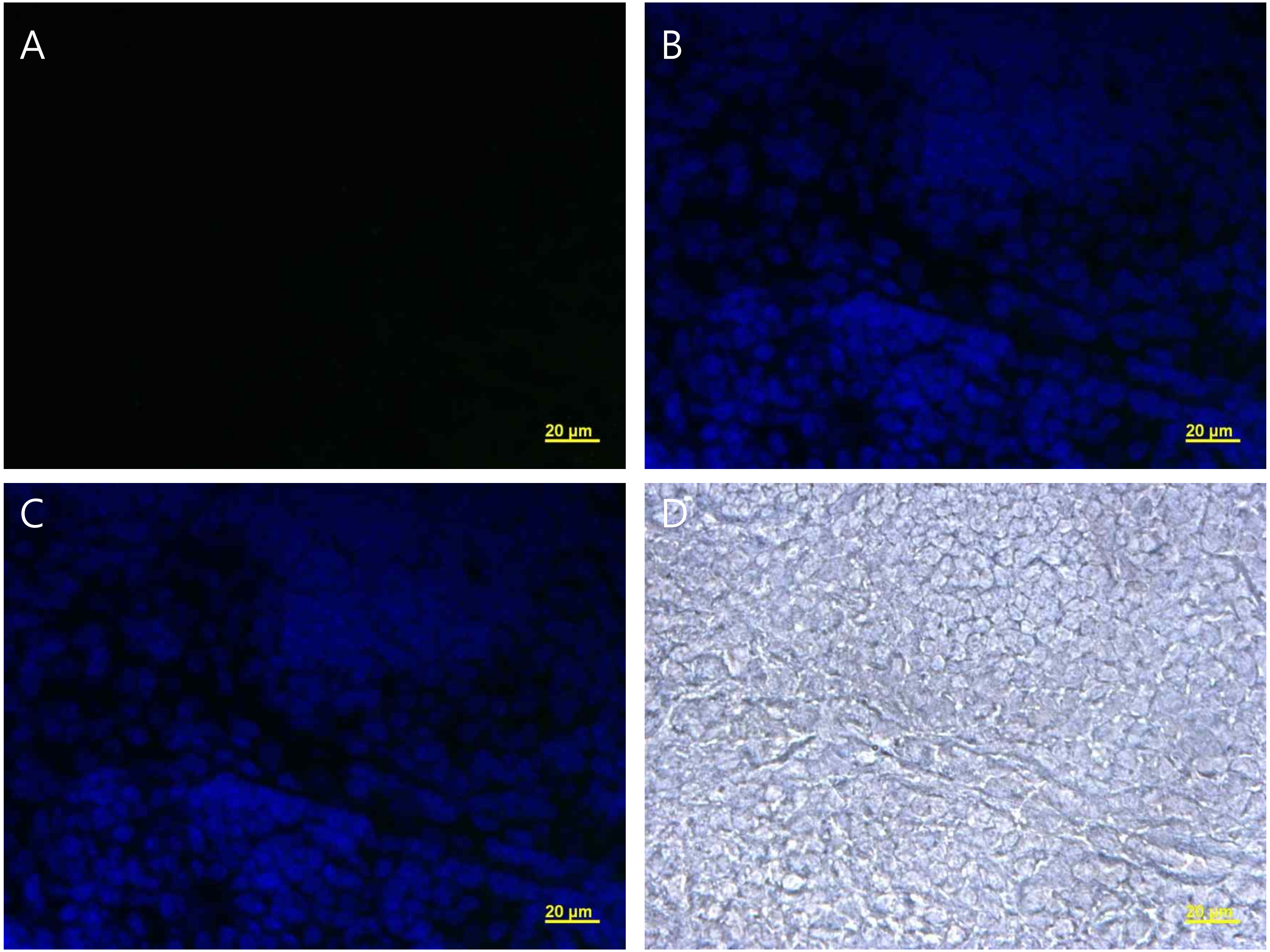

A – CD31

B – DAPI

C – CD31 +DAPI

D – Phase contrast

Fig. 1.4 Primo vessel on the fascia of subcutaneous tumor: CD31

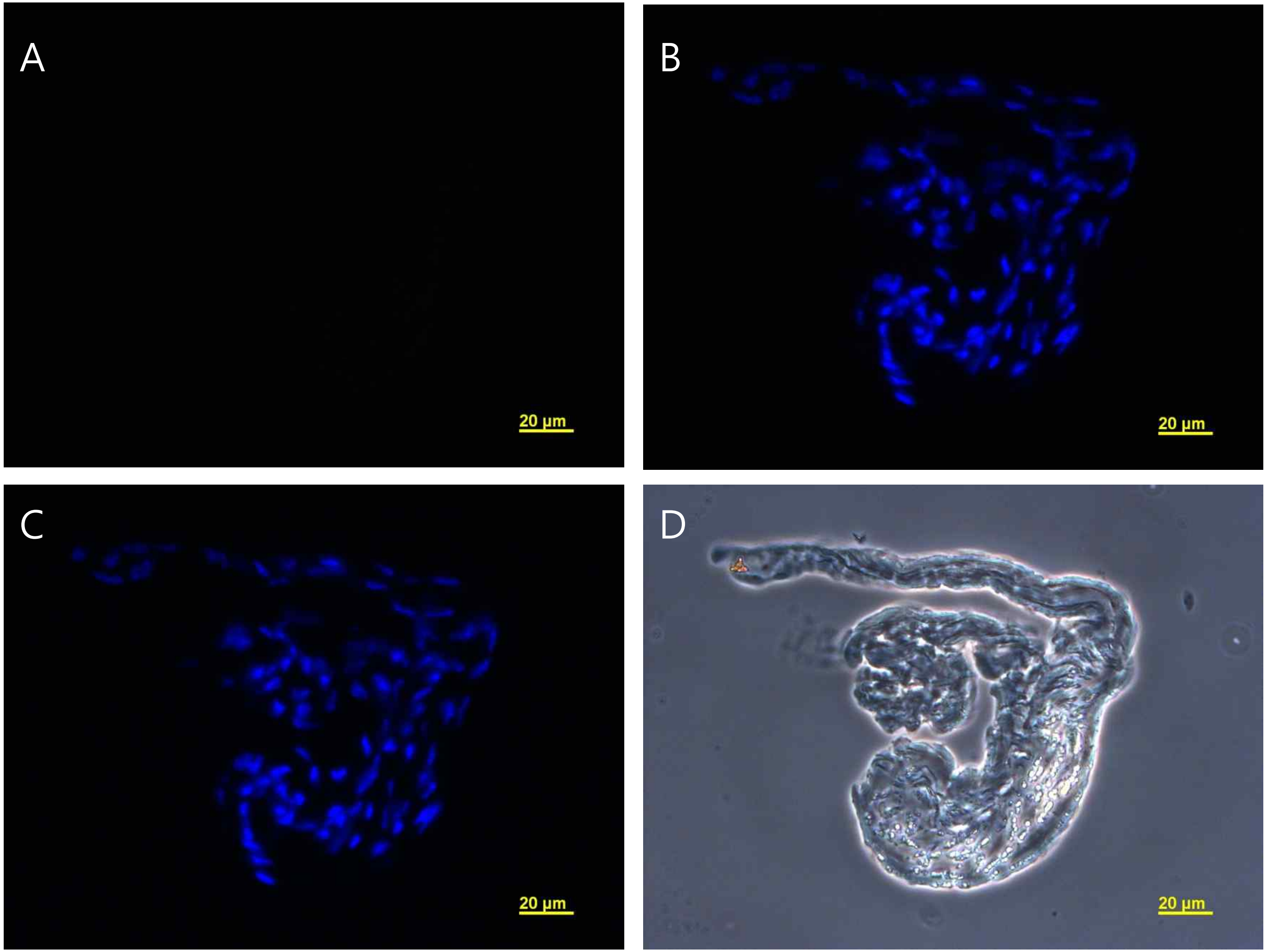

A – CD31
B – DAPI
C – CD31 +DAPI
D – Phase contrast

Fig. 2.1 Artery: LYVE-1

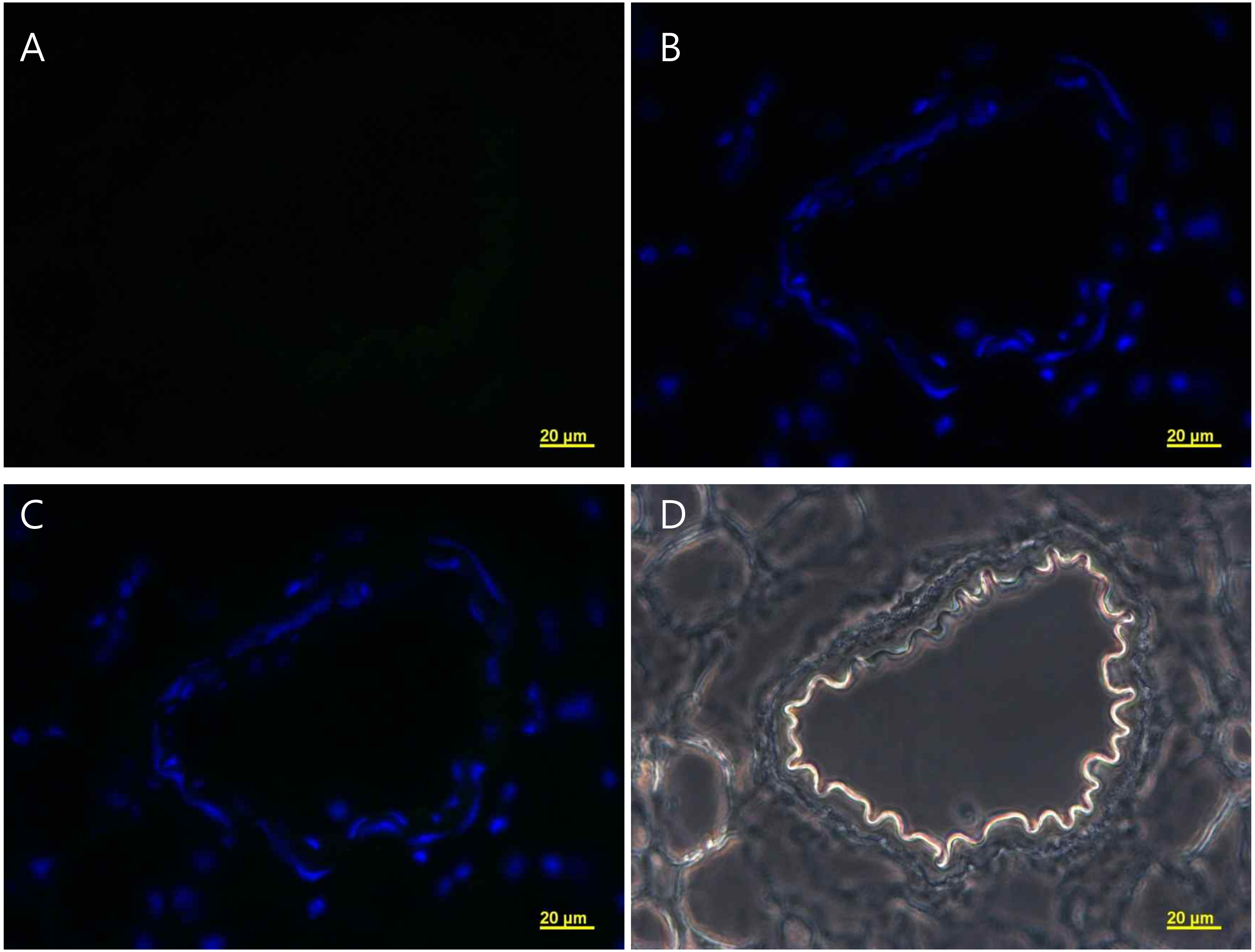

A – LYVE-1
B – DAPI
C – LYVE-1 +DAPI
D – Phase contrast

Fig. 2.2 Vein: LYVE-1

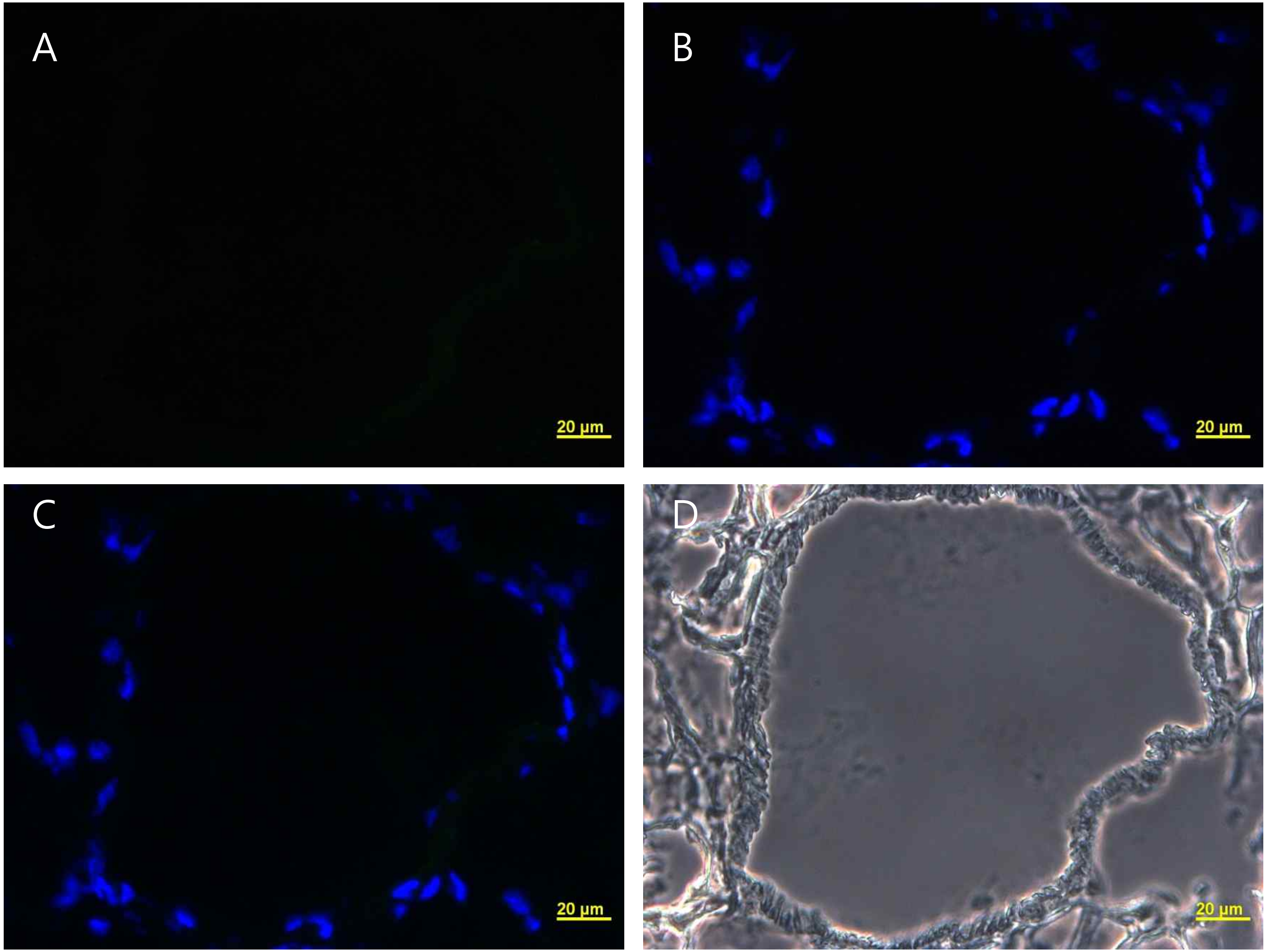

A – LYVE-1
B – DAPI
C – LYVE-1 +DAPI
D – Phase contrast

Fig. 2.3 Lymph node: LYVE-1

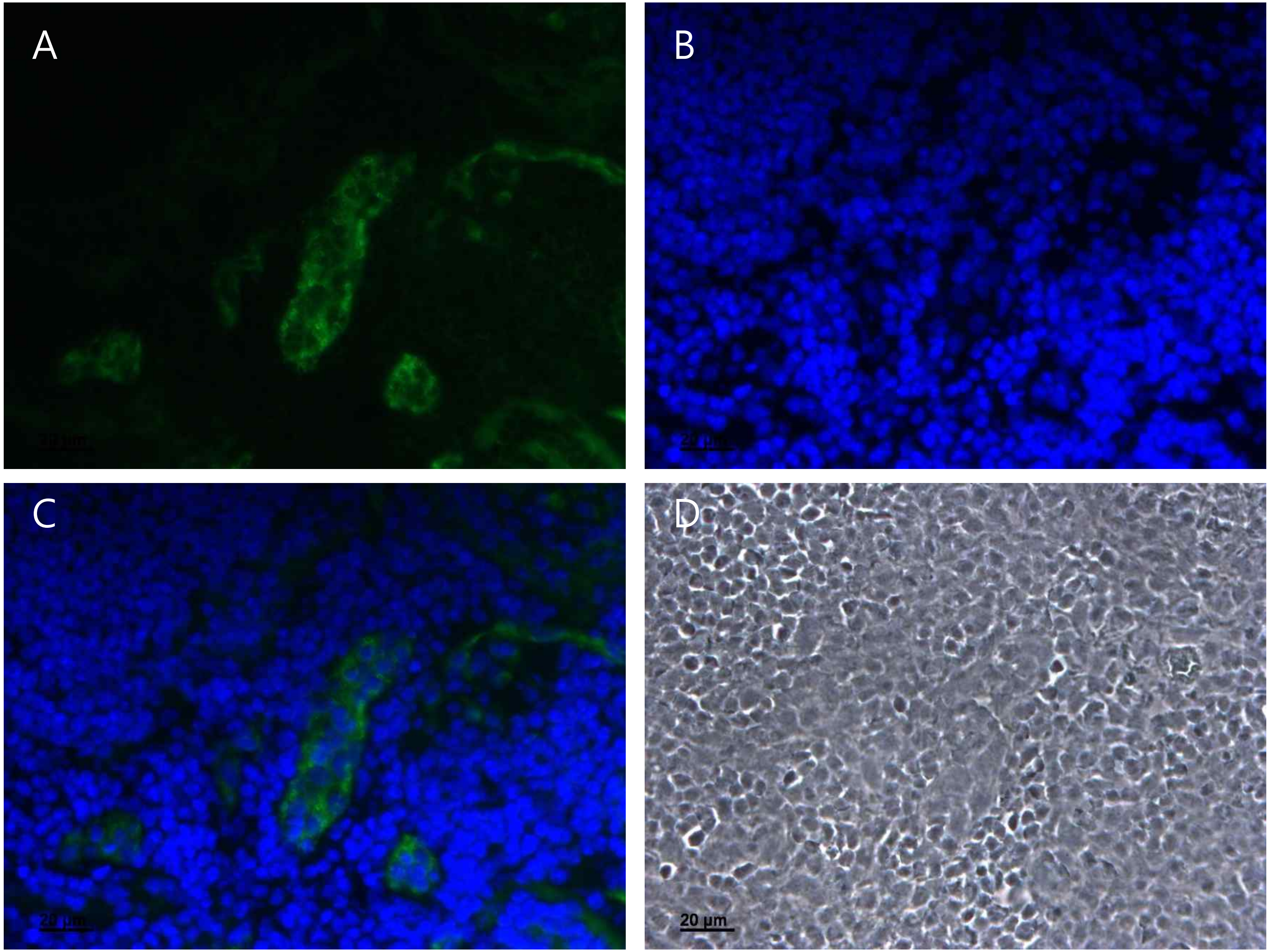

A – LYVE-1
B – DAPI
C – LYVE-1 +DAPI
D – Phase contrast

Fig. 2.4 Primo vessel on the fascia of subcutaneous tumor: LYVE-1

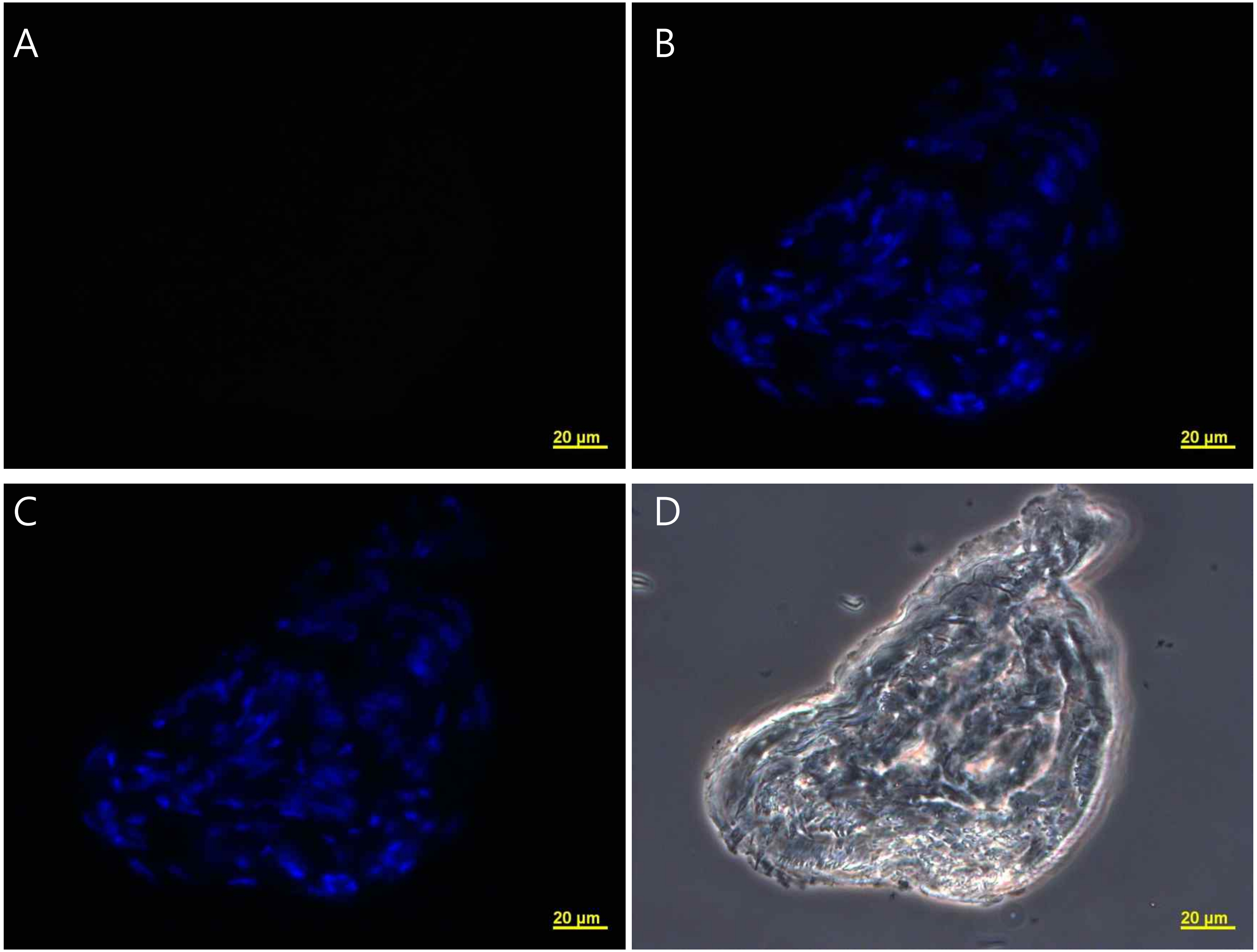

A – LYVE-1
B – DAPI
C – LYVE-1 +DAPI
D – Phase contrast
